# Factors Affecting Mobile Diabetes Monitoring Adoption Among Physicians: Questionnaire Study and Path Model

**DOI:** 10.2196/jmir.2159

**Published:** 2012-12-21

**Authors:** Shintaro Okazaki, José Alberto Castañeda, Silvia Sanz, Jörg Henseler

**Affiliations:** ^1^Department of MarketingCollege of Economics and Business AdministrationUniversidad Autónoma de MadridMadridSpain; ^2^Department of MarketingCollege of Economics and Business AdministrationUniversity of GranadaGranadaSpain; ^3^Department of MarketingFaculty of EconomicsUniversity of ValenciaValenciaSpain; ^4^Institute for Management ResearchRadboud University NijmegenNijmegenNetherlands

**Keywords:** Blood glucose self-monitoring, Diabetes mellitus, Internet, Diabetes self-management, Health informatics, Mobile health, Mobile device, Mobile diabetes monitoring, Path analysis

## Abstract

**Background:**

Patients with type 1 and type 2 diabetes often find it difficult to control their blood glucose level on a daily basis because of distance or physical incapacity. With the increase in Internet-enabled smartphone use, this problem can be resolved by adopting a mobile diabetes monitoring system. Most existing studies have focused on patients’ usability perceptions, whereas little attention has been paid to physicians’ intentions to adopt this technology.

**Objective:**

The aim of the study was to evaluate the perceptions and user acceptance of mobile diabetes monitoring among Japanese physicians.

**Methods:**

A questionnaire survey of physicians was conducted in Japan. The structured questionnaire was prepared in a context of a mobile diabetes monitoring system that controls blood glucose, weight, physical activity, diet, insulin and medication, and blood pressure. Following a thorough description of mobile diabetes monitoring with a graphical image, questions were asked relating to system quality, information quality, service quality, health improvement, ubiquitous control, privacy and security concerns, perceived value, subjective norms, and intention to use mobile diabetes monitoring. The data were analyzed by partial least squares (PLS) path modeling.

**Results:**

In total, 471 physicians participated from 47 prefectures across Japan, of whom 134 were specialized in internal and gastrointestinal medicine. Nine hypotheses were tested with both the total sample and the specialist subsample; results were similar for both samples in terms of statistical significance and the strength of path coefficients. We found that system quality, information quality, and service quality significantly affect overall quality. Overall quality determines the extent to which physicians perceive the value of mobile health monitoring. However, in contrast to our initial predictions, overall quality does not have a significant direct effect on the intention to use mobile diabetes monitoring. With regard to net benefits, both ubiquitous control and health improvement are significant predictors. Net benefits in turn significantly motivate physicians to use mobile health monitoring, and has a strong influence on perceived value. Perceived value and subjective norms are predictors of intention to use. In our sample, concerns over privacy and security risk have no significant effects on intention to use mobile diabetes monitoring. Among the 3 control variables, only age significantly affected intention to use mobile diabetes monitoring, whereas experience and gender were not significant predictors of intention.

**Conclusions:**

Physicians consider perceived value and net benefits as the most important motivators to use mobile diabetes monitoring. Overall quality assessment does affect their intention to use this technology, but only indirectly through perceived value. Net benefits seem to be a strong driver in both a direct and indirect manner, implying that physicians may perceive health improvement with ubiquitous control as a true utility by enhancing cost-effective monitoring, and simultaneously recognize it as a way to create value for their clinical practices.

## Introduction

### Context and Prior Work

The increased need for real-time data management and the advances in mobile communication technology are developing markets for a new form of remote diabetes data management systems [1-3]. Mobile diabetes monitoring (MDM) can provide a more personalized and flexible means of control through which physicians can get immediate medical data and achieve continuous control over patients’ health, while patients can satisfy their desire to receive timely clinical feedback and lower the cost of long-term medical care [4,5]. A recent review of 101 commercial mobile apps found that insulin and medication recording (62%), data export and communication (60%), diet recording (47%), and weight management (43%) are the most prevalent features [1]. The adoption of mobile diabetes monitoring has been examined in several situations. A clinical pilot trial in Austria indicated that a diabetes management system was well accepted by patients and practical for daily usage. A similar application was tested in a randomized and crossover clinical experiment with 10 type 1 diabetic patients aged 21 to 62 years in Spain [6]. The patients showed high acceptability and interest in the system as recorded in usability and utility questionnaires. Martínez-Sarriegui et al [3] tested personal digital assistant (PDA) monitoring with a continuous glucose sensor on 5 diabetic patients in Spain. They found that all patients were satisfied and would recommend the system. In South Korea, a mobile blood glucometer system was tested with 20 elderly patients older than 65 years. Despite complaints related to short battery life and difficulties in operating mobile phones, the patients’ satisfaction was 8.59 of 10 points.

### Significance of the Study

Our literature review indicates that most published studies focus on the use or clinical evaluation of mobile diabetes monitoring from the patients’ perspectives. Empirical research on mobile diabetes monitoring adoption from the physicians’ perspective is almost nonexistent, thus the present study makes a significant contribution to our existing knowledge. Although understanding end users’ (ie, patients) adoption mechanisms is important, the adoption of mobile diabetes monitoring needs to be understood from the operators’ (ie, physicians) perspectives for two reasons. First, unlike typical commercial transactions, patients are not really the customers who choose mobile diabetes monitoring. It is the physicians, along with other health professionals, who make the ultimate clinical decision to introduce information technology (IT) designed to support highly specialized tasks and services [7]. Second, prior research indicates that a close collaboration between physicians and the medical device industry is essential for device innovation [8]. In this view, physicians provide essential knowledge of technology and medical practice that becomes incorporated into new devices. Physicians’ involvement in clinical trials and testing is increasingly important, thus the industry needs profound knowledge about the mechanisms of their adoption behavior.

### Theoretical Background and Model

**Figure 1:**
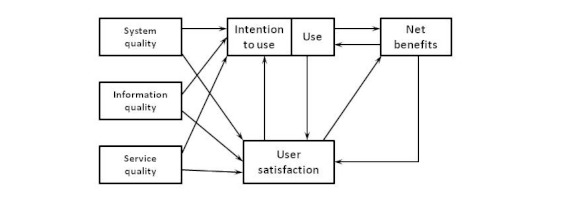
Updated DeLone and McLean Information System (IS) Success Model.

Our theoretical model is based on the updated DeLone and McLean Information System (IS) Success Model, which covers different perspectives of evaluating information systems (Figure 1). DeLone and McLean [9] reviewed the existing definitions of IS success and their corresponding measures, and proposed a basic model. Later, this model was revised by incorporating 7 major variables connected in structural relationships [10]. System quality is defined as the desirable characteristics of an IS, whereas information quality means characteristics of the output offered by the IS. Service quality refers to the quality of the support that system users receive from the IS department and IT support personnel. Net benefits explain the effect an IS has on an individual, group, organization, industry, or society, and influence both the usage extent of the IS and the level of resulted satisfaction [11,12].

Our research closely follows this model for two reasons. First, prior research suggests that the original DeLone and McLean IS Success Model has been one of the most widely cited IS models [11,12]. Second, the updated DeLone and McLean IS Success Model is among the few options that have incorporated quality and net benefits dimensions that we consider to be crucial determinants of mobile diabetes monitoring adoption.

**Figure 2:**
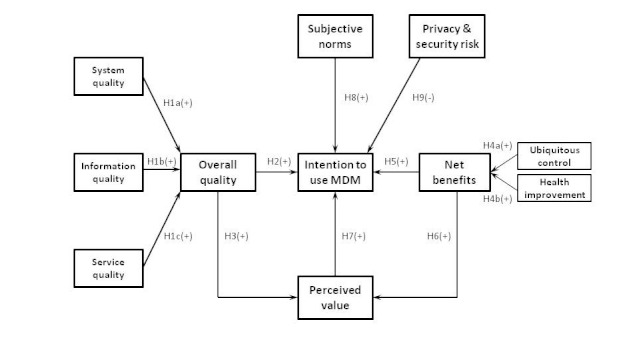
Theoretical model of mobile diabetes monitoring adoption among Japanese physicians showing various hypothesized (H) relationships. A plus sign or minus sign signifies an increase or decrease, respectively, in the dependent variable evoked by an increase in the independent variable (ceteris paribus).

The updated DeLone and McLean IS Success Model can be considered as a conceptual scheme of IS success in a given organization. However, measurements of each variable were only loosely suggested. In an attempt to explicate physicians’ adoption of mobile diabetes monitoring, we extend the updated DeLone and McLean IS Success Model by introducing some new variables. We posit 9 different major hypotheses (H1-H9); our research model is shown in Figure 2.

Overall quality is conceptualized as a second-order hierarchical model in which each first-order factor is a cause of the construct [13]. That is, overall quality is defined as the ultimate result when the IS achieves information, system, and service quality. The reason for this high-order construct is because although the updated DeLone and McLean IS Success Model explains that an information system can be evaluated in terms of information, system, and service quality, no overall quality construct was suggested in the model. The extant research on service quality perception has always embraced such a hierarchical view [14]. We posit that physicians are likely to perform mental calculus by summing up individual quality assessment for information, system, and service. Thus, we hypothesize that system quality will directly and positively affect overall quality of mobile diabetes monitoring (H1a), information quality will directly and positively affect overall quality of mobile diabetes monitoring (H1b), and service quality will directly and positively affect overall quality of mobile diabetes monitoring (H1c).

In the updated DeLone and McLean IS Success Model, each quality dimension affects the subsequent intention to use the IS. By the same token, our model contemplates that overall quality determines the intention to use mobile diabetes monitoring. Actual use is excluded from our model, because the current diffusion level of mobile diabetes monitoring is still in its infancy. For example, industry reports indicate that although the number of home health monitoring devices with embedded cellular connectivity may reach 2.47 million by 2016; only 570,000 were in use worldwide as of 2011 [15]. Nonetheless, we argue that physicians could judge their intention to use mobile diabetes monitoring without real usage experience, because “intention” is an attitude, whereas “use” is a behavior [10]. Therefore, our secondary hypothesis is that overall quality will directly and positively affect intention to use mobile diabetes monitoring (H2).

DeLone and McLean [10] argue that satisfaction is an important success measure of system adoption because it captures the balance of positive and negative impacts of its use. In our model, satisfaction is replaced by perceived value because of the same reason mentioned previously―low penetration of mobile diabetes monitoring. Here, we view value as the performance improvement in functionality, efficiency, productivity, and practicality because of mobile diabetes monitoring adoption as weighed against the associated costs. Because only a limited number of physicians may have actually used mobile diabetes monitoring, the level of satisfaction is not a realistic measure. Instead, we posit that physicians could perceive certain value—perceived trade-off between improved patient care and costs—toward the system. However, such value cannot be perceived without a good understanding of overall quality. For example, within the general diabetes treatment program, the impact of mobile diabetes monitoring on patients cannot be viewed as valuable unless physicians fully evaluate the overall performance quality it delivers to the clinical practitioners. Thus, our third hypothesis is that overall quality will directly and positively affect perceived value (H3).

DeLone and McLean [10] suggest that net benefits must be determined by the context and objectives of the IS investment by asking the following questions: What qualifies as a “benefit”? For whom? And at what level of analysis? In this regard, we conceptualize net benefits as a composite effect of two variables: ubiquitous control and health improvement. Ubiquitous control is defined as flexible patient care without time and place restriction. Ubiquity has been suggested to be the most important utility of mobile device [16], and thus the heart of mobile diabetes monitoring benefits. Health improvement in this context encompasses the clinical advantages physicians could achieve through the use of mobile diabetes monitoring, which have been suggested by several trial experiments [3,6,17]. We therefore expect that ubiquitous control will directly and positively affect net benefits (H4a), and health improvement will directly and positively affect net benefits (H4b).

Prior research measures net benefits as improvements in job performance and finds that they significantly impact intention to use knowledge-management systems [18,19]. Similar findings have been reported in IS literature [12], thus leading us to hypothesize that net benefits will directly and positively affect intention to use mobile diabetes monitoring (H5).

If physicians indeed perceive important benefits derived from mobile diabetes monitoring (ie, ubiquitous control and health improvement), they may ultimately see an opportunity to create value for their clinical practice. Prior research suggests that such value creation would lead to a stronger intention to adopt IS [20]. Thus, the net benefits will directly and positively affect perceived value (H6), and perceived value will directly and positively affect intention to use mobile diabetes monitoring (H7).

Next, theory of planned behavior (TPB) states that behavioral intentions are determined by three primary dimensions: attitude, subjective norm, and perceived behavioral control. All three factors are influenced by a set of cognitive beliefs about the innovation and their respective importance. Of special interest to the present study, subjective norm can be defined as a “person’s perception that most people who are important to him think he should or should not perform the behavior in question” [21]. Prior research based on TPB finds that subjective norm has the strongest effect on physicians’ behavioral intentions to share knowledge [22,23]. This finding implies that peer influence may play a central role in the adoption of mobile diabetes monitoring, leading to the hypothesis that subjective norms will directly and positively affect intention to use mobile diabetes monitoring (H8).

Finally, a general concern for privacy and security risk in mobile commerce may be applicable to the adoption of mobile diabetes monitoring. In fact, both industry practitioners and scholars have debated potential risks involved in electronic medical records [24-26]. Thus, the treatment of personal data and data security can be a negative driver of usage intention. Therefore, we posit that privacy and security risk will directly and negatively affect intention to use mobile diabetes monitoring (H9).

### Purpose

The purpose of our empirical survey is to validate the explanatory power of our research model, which is a theoretical extension of the updated DeLone and McLean IS Success Model. A set of key attitudinal and perceptual factors of mobile diabetes monitoring adoption are assessed from the physicians’ point of view.

## Methods

### Site of the Study

Japan served as the site for this study for several reasons. First, it has one of the highest mobile broadband penetration rates: In 2011, there were 122 million 3G subscribers with 95% penetration rate [27]. Second, Japan is the country with the eighth-largest number of diabetes patients worldwide in 2010, with approximately 7.3 million adults aged 20 to 79 years [28]. According to the World Health Organization (WHO), this number is expected to reach 8.9 million by 2030 [29]. Thus, an innovative treatment approach for diabetes would draw much attention from physicians. Third, with aging of the Japanese population, the health care costs associated with chronic diseases are becoming a serious burden in Japan’s social security system. To address this issue, there have been various strategic initiatives, of which the first two were targeted toward diabetes and depression [29]. Fourth, the direct health care costs of diabetes are due to increased costs associated with hospital admissions and outpatient visits, as well as the costs of medications. Oishi and colleagues [30] performed a study in 2003, examining the time and costs in caring for patients with newly identified type 2 diabetes and other lifestyle diseases in Japan. The mean number of monthly doctor visits was similar for newly diagnosed patients with diabetes and for patients with hypertension and/or hyperlipidemia, but the total time of these visits for patients with diabetes was greater [30]. Thus, the adoption of mobile diabetes monitoring could significantly reduce potential costs associated with keeping these patients’ diabetes under control. All these reasons ensure the ecological validity of the study, and thus justify the use of Japan as the site of the study.

### Definition of Mobile Diabetes Monitoring

This study defines mobile diabetes monitoring as a system of self-monitoring blood glucose in diabetic patients by means of 3G-enabled mobile device. Typically, mobile diabetes monitoring enables the following functions: (1) self-monitoring of blood glucose, weight, physical activity, diet, insulin and medication, and blood pressure; (2) disease-related data export and physician-patient communication; and (3) synchronization with personal health record systems at the hospital’s information hub. This technical definition is consistent with prior research [1], and leading Japanese medical informatics laboratories and firms (eg, University of Tsukuba, Fujitsu) developed a similar system [31,32].

### Questionnaire

A questionnaire survey was conducted in Japan. At the beginning of the questionnaire, we asked the medical specialty of the respondent and their level of clinical experience (in years). We then showed a graphical image of a mobile-based blood glucose self-monitoring system developed by the University of Tsukuba [32]. In addition, a detailed description of the system purpose, functions, and usage procedures were provided. We asked whether the respondents had used mobile diabetes monitoring. If the answer was affirmative, we then asked them to rate each item according to their usage experience; otherwise, their assessments were based on the description provided in the questionnaire. In the next section, we listed the questions related to the model constructs explained in the previous section: system quality, information quality, service quality, perceived health improvement, privacy and security concerns, perceived value for medical control, subjective norms, ubiquitous control, and intention to use mobile diabetes monitoring. Construct measures were adapted from previous research in information systems, health care, and consumer behavior [14,16,33-43]. All constructs were measured by multiple-item scales with a 7-point Likert scale, except perceived value that was measured by a 7-point semantic differential scale. At the end of the questionnaire, some demographic questions, such as age, gender, and geographical area (ie, prefecture), along with other relevant questions (eg, usage experience and frequency of Internet and mobile device use), were included. All constructs’ measures used in the present study are listed in Multimedia Appendix 1.

### Participant Recruitment

The survey participants were recruited by a professional research agency in Japan. The agency posted a recruitment notification on its website. As a result, 590 physicians signed up to participate in the survey. As an incentive, the respondents were paid 5000 yen (approximately US $60) for their participation. The survey website was created and the invitation was sent to the participants. Although our sampling was not probabilistic (ie, judgment sample), the respondents were drawn from 47 prefectures in Japan, and thus not clustered in certain regions of Japan.

### Analytical Approach

We applied partial least squares (PLS) path modeling as implemented in SmartPLS 2.0 M3 [44] as means of statistical analysis. PLS has found widespread use in technology adoption and information systems literature [45], primarily because of its suitability for exploratory studies in early stages of research when the focus lies on saturated, prediction-oriented models. We used the factor-weighting scheme as our inner weighting scheme because of its robustness [46]. Two of our constructs, overall quality and net benefits, were modeled as second-order constructs by using the repeated-indicators approach [45]. We applied bootstrapping with 5000 bootstrap samples to obtain inference statistics.

We performed a PLS analysis with two nested samples. First, we conducted the PLS analysis with the total sample. The reason for this is that mobile-based monitoring systems can be applied not only to diabetes, but also to epidemiology of other medical fields. Thus, the inclusion of other medical experts increases external validity of our proposed model. Moreover, the reliability and validity of the measures were assessed with the total sample, based on the criteria formulated by Ringle et al [45]. The internal consistency reliability was estimated using Cronbach alpha and Jöreskog’s rho. Convergent validity was assessed by using the average variance extracted (AVE). We relied on the Fornell-Larcker criterion [47] to assess discriminant validity. Second, because diabetes is normally treated by physicians of internal medicine and gastrointestinal medicine, we limited the sample only to those respondents specialized in these areas. Gastrointestinal symptoms are reportedly common in diabetes [48]. This validation with the specialist subsample should reflect more accurate perceptual, attitudinal, and behavioral responses from the physicians specialized in this specific disease category.

## Results

### Sample Characteristics

During August 2011, 505 physicians responded to the survey. However, there were 34 responses with exaggerated extremity preferences (all 1s or 7s). They were considered to be due to extreme response bias, and were eliminated from the final dataset. Therefore, the total usable sample size was 471, with an effective response rate of 79.8%.

The respondents belonged to diverse specialties (Table 1), including general medicine (20.6%), surgery (10.4%), and gastrointestinal medicine (7.9%), among others. The average clinical experience of the respondents was 19.3 years. Approximately 87% and 13% of the respondents were male and female, respectively, aged 25 to 65 years. A cross-tabulation of sex and age group is shown in Table 2.

**Table 1:** Medical specialties of all respondents (N=471) and those respondents in the subspecialties of general internal medicine and gastrointestinal medicine (n=134) to a survey in Japan about mobile diabetes monitoring.

Specialty	Total sample		Specialist subsample	
	n	%	n	%
General internal medicine	97	20.6	97	72.4
Psychosomatic medicine	1	0.2	0	0
Respiratory internal medicine	7	1.5	0	0
Gastrointestinal medicine	37	7.9	37	27.6
Cardiovascular medicine	21	4.5	0	0
Neurology	13	2.8	0	0
Nephrology	12	2.5	0	0
Hematology	4	0.8	0	0
Psychiatry	28	5.9	0	0
Surgery	49	10.4	0	0
Obstetrics and gynecology	14	3.0	0	0
Ophthalmology	19	4.0	0	0
Otorhinolaryngology	13	2.8	0	0
Dermatology	15	3.2	0	0
Urology	9	1.9	0	0
Radiology	17	3.6	0	0
Anesthesiology	25	5.3	0	0
Others	90	19.1	0	0

**Table 2:** Age and sex distribution of respondents to survey in Japan about mobile diabetes monitoring.

Age	Sex, n (%)		Total, n (%) n=471
	Male n=410	Female n=61	
20-29 years	9 (2.2)	11 (18.0)	20 (4.2)
30-39 years	74 (18.0)	24 (39.3)	98 (20.8)
40-49 years	174 (42.4)	18 (29.5)	192 (40.8)
50-59 years	125 (30.5)	6 (9.8)	131 (27.8)
≥ 60 years	28 (6.8)	2 (3.3)	30 (6.4)

In terms of personal use, approximately 42% of the respondents accessed the Internet with their mobile device on a daily basis; 9% did so more than 10 times a day. By contrast, 26.9% did not access the Internet with mobile device. On average, the respondents used the Internet with their mobile device for approximately 7 years. In terms of professional use, approximately 30% of the respondents used the Internet for their patient care or other clinical practices. With regard to mobile diabetes monitoring, only 0.8% of the respondents actually used mobile diabetes monitoring previously. Although 25.9% were aware of its functions but did not use mobile diabetes monitoring, 73.2% were not well informed.

### Measurement Validation

All our constructs exhibit sufficient levels of internal consistency reliability, exceeding the recommended threshold of .70 [49]. All AVE values were above the critical value of .50 [47], which indicates that all constructs are unidimensional, thus implying convergent validity. In Table 3, we report Cronbach alpha, Jöreskog’s rho, and the AVE.

Table 4 shows the construct correlations as well as the square root of the AVE as the basis for assessment of discriminant validity. Comparing the square root of each construct’s AVE with its largest absolute correlation shows that the Fornell-Larcker criterion is met. Therefore, discriminant validity can also be confirmed.

**Table 3:** Quality indicators of the constructs, including Cronbach's alpha, Jöreskog’s rho, and average variance extracted (AVE).

Construct	Number of items	Cronbach's alpha	Jöreskog’s rho	AVE
Age	1	―	―	―
Experience	1	―	―	―
Gender	1	―	―	―
Health improvement	4	.90	.93	.77
Information quality	11	.96	.97	.73
Intention to use mobile diabetes monitoring	3	.92	.95	.87
Perceived value	8	.96	.96	.77
Security/privacy concerns	8	.96	.97	.78
Service quality	9	.96	.96	.74
Subjective norms	3	.94	.96	.89
System quality	10	.91	.93	.56
Ubiquitous control	6	.94	.96	.78

**Table 4:** Construct correlations and square root of the AVE.

Construct		Construct correlations^a^											
		1	2	3	4	5	6	7	8	9	10	11	12
1	Age	―											
2	Experience	.00	―										
3	Gender	–.30	.08	―									
4	Health improvement	.04	–.06	–.04	*.88*								
5	Information quality	.00	–.02	–.06	.80	*.85*							
6	Intention to use	–.08	–.03	–.04	.64	.70	*.93*						
7	Perceived value	.01	–.07	–.07	.72	.81	.72	*.87*					
8	Security/privacy concerns	–.05	–.07	.03	.17	.30	.20	.24	*.88*				
9	Service quality	.08	.00	–.02	.70	.66	.49	.62	.04	*.86*			
10	Subjective norms	.06	.00	–.10	.67	.70	.76	.72	.15	.60	*.94*		
11	System quality	.09	.00	–.07	.69	.75	.57	.71	.13	.74	.64	*.75*	
12	Ubiquitous control	.08	–.05	–.04	.71	.78	.72	.77	.27	.60	.77	.69	*.88*

^a^ Diagonal elements in italics are the square root of the construct’s AVE (all other elements are correlations between the constructs).

### Model Validation with Total Sample

**Figure 3:**
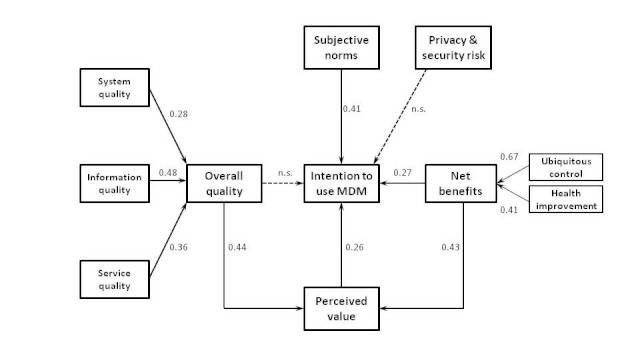
Partial least squares (PLS) analysis results of the theoretical model of mobile diabetes monitoring adoption among Japanese physicians. The numbers indicate standardized beta coefficients.

The results of the PLS analysis on the total sample are shown in Figure 3. The model estimates largely confirm our conceptual model. System quality, information quality, and service quality all contribute significantly to the overall quality. Remarkably, the indirect effect of overall quality on the intention to use mobile diabetes monitoring is positive (indirect effect = 0.11) and significant (*P* < .001). However, the total effect of overall quality on the intention to use mobile diabetes monitoring is not significant (total effect = 0.05, *P* = .21). As the indirect effect of overall quality already suggests, both the effect of overall quality on perceived value and the effect of perceived value on the intention to use mobile diabetes monitoring are significant. Ubiquitous control and health improvement contribute significantly to the formation of net benefits. Net benefits, in turn, have significant positive effects on the intention to use mobile diabetes monitoring and perceived value. Also, the indirect effect (indirect effect = 0.11, *P* < .001) and the total effect (total effect = 0.38, *P* < .001) of net benefits on the intention to use mobile diabetes monitoring are significant. As anticipated, subjective norms have a significant positive effect on the intention to use mobile diabetes monitoring. Finally, privacy and security risk does not have a significant effect on the intention to use mobile diabetes monitoring. The control variables tested (age, experience, and gender of the respondents) did not produce any effect with one exception: the negative influence of the age of physicians on intention to use mobile diabetes monitoring. The older respondents have a significantly lower intention to use mobile diabetes monitoring than the younger respondents, suggesting that the younger the physicians, the more likely they will be to adopt and use mobile diabetes monitoring. This effect is likely because of the widely documented adverse relationship between age and new technology acceptance.

### Model Validation with Specialist Subsample

Table 5 lists the results from the PLS analysis of the specialist (internal and gastrointestinal medicine) subsample juxtaposed with those from the total sample validation. As far as statistical significance is concerned, no discrepancy was found between the two results. Except for two paths (ie, overall quality → intention to use mobile diabetes monitoring, and privacy and security risk → intention to use mobile diabetes monitoring), all hypothesized relationships were supported. Furthermore, the magnitudes of standardized beta coefficients were also very similar.

### Hypotheses Testing

Hypotheses testing results are summarized in the far right column of Table 5. Based on the results from both the total sample and the specialist subsample, we could confirm that all but two hypotheses are supported by our data. More specifically, system quality, information quality, and service quality significantly affect overall quality, providing support for hypotheses 1, 2, and 3. Our results also indicate that overall quality determines the extent to which physicians perceive the value of mobile health monitoring. However, in contrast to our initial predictions, overall quality does not have a significant direct effect on the intention to use mobile diabetes monitoring. Thus, hypothesis 2 does not gain support but hypothesis 3 does. With regard to net benefits, both ubiquitous control and health improvement are significant predictors, which ring true for hypotheses 4a and 4b. Net benefits in turn significantly motivate physicians to use mobile health monitoring, while exercise strong influence on perceived value. Thus, both hypotheses 5 and 6 are supported. Perceived value is found to be a strong predictor of intention to use, which provides support for hypothesis 7. In the same token, as predicted in hypothesis 8, subjective norms significantly affect intention to use. Finally, concerns over privacy and security risk have no significant effects on intention to use mobile diabetes monitoring. Thus, hypothesis 9 is not supported.

**Table 5:** Summary of partial least squares (PLS) estimation from the total sample and the specialist (internal and gastrointestinal medicine) subsample.

Hypotheses	Path^a^	Total sample (n=471)		Specialist subsample (n=134)		Hypothesis testing results
		Beta coefficients	*P*	Beta coefficients	*P*	
Hypothesis 1a	System quality → overall quality (+)	.28	< .001	.30	< .001	Supported
Hypothesis 1b	Information quality → overall quality (+)	.48	< .001	.46	< .001	Supported
Hypothesis 1c	Service quality → overall quality (+)	.36	< .001	.33	< .001	Supported
Hypothesis 2	Overall quality → intention to use (+)	−.07	.15	−.07	.44	Unsupported
Hypothesis 3	Overall quality → perceived value (+)	.44	< .001	.47	< .001	Supported
Hypothesis 4a	Ubiquitous control → net benefits (+)	.67	< .001	.65	< .001	Supported
Hypothesis 4b	Health improvement → net benefits (+)	.41	< .001	.40	< .001	Supported
Hypothesis 5	Net benefits → intention to use (+)	.27	< .001	.20	< .05	Supported
Hypothesis 6	Net benefits → perceived value (+)	.43	< .001	.41	< .001	Supported
Hypothesis 7	Perceived value → intention to use (+)	.26	< .001	.28	.01	Supported
Hypothesis 8	Subjective norms → intention to use (+)	.41	< .001	.54	< .001	Supported
Hypothesis 9	Privacy and security risk → intention to use (−)	.02	.22	.09	.09	Unsupported
Control variable	Age → intention to use	−.12	< .001	−.09	.06	n.a.^b^
Control variable	Experience → intention to use	.00	.48	.04	.36	n.a.^b^
Control variable	Gender → intention to use	−.01	.33	.01	.89	n.a.^b^

^a^ The plus (+) or minus (–) sign in parentheses denotes whether a positive or negative effect is anticipated.

^b^ n.a. = not applicable.

## Discussion

### Principal Results

Our proposed model was successfully validated by the total sample and also by the specialist subsample. The statistical significance and the strength of standardized coefficients were almost identical for all the hypothesized paths. Therefore, for the sake of simplification, our principal results are described based on the total sample validation.

In terms of direct effects, physicians’ intention to use mobile diabetes monitoring is primarily influenced by net benefits, perceived value, and subjective norms. Our PLS results indicate that the multivariate coefficient of determination (*R* ^*2*^) value of intention to use mobile diabetes monitoring was 0.67 (*R* ^*2*^ = 0.82 for the specialist subsample), indicating that more than two-thirds of the variance of this construct can be explained by the model. This value can be described as “substantial” according to Chin [50].

Net benefits not only have a direct effect on intention, but also an indirect effect through perceived value. We thus find evidence for a partial mediation. The hypothesized direct effect of overall quality on intention cannot be confirmed, but we find a significant positive indirect effect. More specifically, although neither the direct effect nor the total effect of overall quality on the intention to use mobile diabetes monitoring is significant, the indirect effect of overall quality on the intention to use mobile diabetes monitoring is significant. Given the correlation between overall quality and intention to use mobile diabetes monitoring (total *R* ^*2*^ = 0.67; specialist subsample *R* ^*2*^ = 0.70), the influence of overall quality on usage intention appears to be fully mediated by perceived value. This means that overall quality contributes to forming the intention to use mobile diabetes monitoring only if an increase in overall quality is perceived as value added.

Privacy and security concerns seem to be negligible in terms of their impact on the intention to use mobile diabetes monitoring. This is surprising given that there is much documented evidence about such concerns in wireless medical devices [51]. One possible explanation is that perhaps the respondents of this study might have been more experienced in the use of innovative medical devices. Prior research indicates that frequent and experienced users of electronic health records were significantly less concerned about privacy and security than nonusers [52]. On the other hand, there seems to exist evidence that, compared with general business practitioners, many physicians are not particularly technology literate, despite their highly demanding educational and specialized training [7]. This lack of computer literacy may have made them fail to acknowledge the severity of privacy and security concerns in mobile diabetes monitoring. We deem the latter to be a more reasonable and realistic explanation.

### Limitations

The sample size of physicians specialized in internal medicine or related specialties was modest. Future studies should ensure a larger sample to increase the generalizability of the findings. Similarly, although the sample consisted of physicians all across Japan (ie, 47 prefectures), the selection was not probabilistic; thus, it may not accurately reflect the entire Japanese medical community. Third, we did not examine how physicians’ personal propensity to use new information and communication technology affects the model variables, which was far beyond the scope of our study. Because of the preceding reasons, this study should be considered as an initial stepping-stone and any generalization of the results should be done with caution.

### Conclusions and Practical Implications

Physicians from a wide range of clinical expertise most valued perceived value as a mediator of the effects exercised by both overall quality and net benefits over intention to use mobile diabetes monitoring. This fact seems even more significant given the absence of direct effect of overall quality on intention to use mobile diabetes monitoring because physicians apparently seem to weigh exactly what kind of value they can get out of the system. In health care, value is defined as the patient health outcomes achieved relative to the inputs (or cost) required [53]. Because net benefits already take into account health outcome (ie, health improvement) and device utility (ie, ubiquitous control), our perceived value seems to crystallize the importance of the output-input relationship derived from the mobile diabetes monitoring. Prior research suggests that the power of quality improvement to drive down costs is greater in health care than in any other field [53]. Thus, our results may suggest that physicians tend to look into the efficiency and cost-effectiveness of mobile diabetes monitoring before forming their usage intention. This implies that the medical device industry should be increasingly keen on these aspects in marketing wireless medical monitoring systems.

The impact of subjective norms on intention to use mobile diabetes monitoring was solid, but this finding seems somewhat contradictory to the literature. Prior research on telemedicine finds no impact of subjective norms on intention, suggesting that physicians may value their own assessments over others’ opinions and suggestions [7]. Similarly, the effects of subjective norms on intention to use clinical guidelines were reported to be much weaker among physicians than among nurses and other clinical professionals [54]. Our interpretation is that because many physicians are not particularly computer- or Internet-literate, they may rely more on their peers’ or colleagues’ help and suggestions regarding mobile diabetes monitoring adoption (ie, medical and IS adoption decisions), whereas they tend to be more independent or self-reliant for other knowledge-based practices (ie, purely medical decisions). Given the solid effects of subjective norms on intention to use mobile diabetes monitoring, it would be wise to organize workshops, seminars, or informative sessions so that knowledge and familiarity could be disseminated through word-of-mouth among peers and colleagues.

Finally, this study would help physicians understand the usefulness of mobile-based health care systems. Diabetes is a leading disease in most developed countries and requires constant and continuous monitoring. For example, according to the US Department of Health and Human Services, diabetes mellitus was listed as the seventh leading cause of death in 2010 [55]. Mobile technology could encourage diabetic patients to lead healthier lives and facilitate earlier discovery of life-threatening symptoms.

## Multimedia Appendix 1

Construct measures used in the study.

## Abbreviations

AVE: average variance extracted
IS: information system
IT: information technology
MDM: mobile diabetes monitoring
PDA: personal digital assistant
PLS: partial least squares
TPB: theory of planned behavior
