# Supplementary material for: Factors Affecting Mobile Diabetes Monitoring Adoption Among Physicians: Questionnaire Study and Path Model
Source: J Med Internet Res. 2012 Dec 21;14(6):e183. doi: 10.2196/jmir.2159 (PMC3558050; doi:10.2196/jmir.2159)
Supplement: Supplementary file 1 [file jmir_v14i6e183_app1.pdf]

Constructs	Dimension/Source	Items
System quality	Gorla et al. (2010) [34]	Our information systems are easy to learn.<br>Our information systems are equipped with only useful features and functions.<br>Our information systems are flexible to adapt/make changes easily.
	Parasuraman et al. (2005) [14]	This service is always available for business.<br>This service launches and runs right away.<br>This service does not crash.<br>This monitoring service does not freeze after I enter my inquiry or retrieve information.
	Hong et al. (2001) [35]	The commands of this monitoring service are well depicted by buttons and symbols.<br>The layout of this monitoring service screen is clear and consistent.<br>The design of this monitoring service is easy to use or operate.
Information quality	Lee et al. (2002) [39]	The information we obtain from this service includes all necessary values.<br>The information we obtain from this service is sufficiently complete for our needs.<br>The information we obtain from this service covers the needs of our tasks.<br>The information we obtain from this service has sufficient breadth and depth for our task.
	Lee et al. (2002) [39]	The information we obtain from this service is easy to understand.<br>It is easy to interpret the information we obtain from this service.<br>The information from this service is easy to comprehend.<br>The meaning of the information we obtain from this service is easy to understand.
	Tan and Chou (2008) [41]	When using this monitoring service, I believe the service/information I receive is specific to my work.<br>The information I received from this service can be customized for my needs.

		When using this monitoring service, I only receive relevant information.
Service quality	Chen (2010) [33]	Our information system specialists give us personal attention.<br>Our information system specialists have our best interests at heart.<br>Our information system specialists understand our specific needs for the patients' information.
	Chen (2010) [33]	Our information system specialists give us prompt service.<br>Our information system specialists tell us exactly when services will be performed.<br>Our information system specialists are never too busy to respond to our requests.
	Chen (2010) [33], Wu and Wang (2006) [19]	When I have a problem with the system, our information system specialists will show a sincere interest in solving it.<br>Our information system specialists provide their services when promised.<br>Our information system specialists are dependable.
Health improvement	Landon et al. (2007) [38], Kelley et al. (2011) [36]	With this monitoring system, most recent blood pressure readings can/will be improved for patients with diabetes or renal failure.<br>With this monitoring system, most recent glucose levels can/will be stabilized for patients with diabetes or renal failure.<br>With this monitoring system, most recent glucose levels can/will be improved for patients with diabetes or renal failure.<br>With this monitoring system, urgent care visits of my patients with diabetes or renal failure have decreased.
Privacy	Lwin et al. (2007) [40]	I am concerned that my patients' personal data may be used for purposes other than monitoring their health.<br>I am concerned about how my patients' online privacy is protected on this monitoring system.<br>I am concerned about the fact that this monitoring system might know/track my patients' personal data.<br>I am concerned about this monitoring system sharing my patients'

		personal information with other parties.
Security	Original items	For this kind of system, there should be no security hole.<br>When we implement this kind of system, I am concerned with unauthorized third-party access to personal information.<br>In using this kind of system, we should make sure that no unauthorized modification could occur to our data.<br>In using this kind of system, I worry that a malicious third party could hack the system.
Perceived value	Kleijnen et al. (2007) [37]	Evaluate the value of mobile health monitoring system on the following dimensions:<br><br>Ineffective–effective<br>Not functional–functional<br>Impractical–practical<br>Useless–useful<br>Not sensible–sensible<br>Inefficient–efficient<br>Unproductive–productive<br>Bad–good
Intention to use	Taylor and Todd (1997) [42]	I intend to use this monitoring system if I am asked to do so.<br>I intend to use this monitoring system when it is available at my work.<br>I intend to use this monitoring system in the near future.
Subjective norms	Taylor and Todd (1997) [42]	My mentors who influence my behavior would think that I should use this monitoring system.<br>My colleagues who are important to me would think that I should use this monitoring system.<br>People whose expertise I trust would think that I should use this monitoring system.
Ubiquitous control	Okazaki et al. (2009) [16]	*Time flexibility*<br>I can obtain the patients' health information from the system on

		time.<br>When I try to follow up and update the patients’ health condition, this monitoring service responds quickly.<br>The monitoring service I use makes the patients’ health information immediately accessible.<br>*Spatial flexibility*<br>Using mobile Internet enables me to find information at any place.<br>Browsing mobile Internet gives me an ability to overcome spatial limitations.<br>Browsing mobile Internet sites fits any location, wherever I go.
